# Is Extirpation of the Cancerous Uterus a Justifiable Operation?

**Published:** 1891-09

**Authors:** John H. McIntyre

**Affiliations:** 614 Olive street; St. Louis, Mo.


### IS EXTIRPATION OF THE CANCEROUS UTERUS A JUSTIFIABLE OPERATION?

BY JOHN H. MCINTYRE, A. M., M. D., ST. LOUIS, MO.*

According to observations made by Gusserow, Lebert, Seifert and others, the life of women affected with cancer of the womb from its first manifestation is about twenty months. It is not surprising therefore, that a few operators attempted to gain a greater likelihood of eradicating the disease by the removal of the entire uterus either through the vagina, or by abdominal section.

From statistics to which I have access, I find that the can-

*Read before the Hodgen District Medical Society, Nevada, Mo., July 9, 1891.

cerous uterus has been extirpated about five hundred times; approximately one hundred and fifty by abdominal section, and three hundred and fifty by the vagina.

On account of the high and frightful mortality resulting from the abdominal operation, not less than 72 per cent., it has been abandoned, except in a very small number of cases where the vaginal method is not feasible. I find but a single case reported of a woman subjected to this method of operation who lived over one year, most of them died in less than six months, and scarcely any lived a year.

Vaginal hysterictoimes, while not so fatal as abdominal, yet gives such a high rate of mortality as to be entirely unjustifiable; of seventeen cases reported in a large western city, nine of the cases were promptly fatal.

No less bold, skilful and successful operator than Mr. I. Knowlesly Thornton, of London, says: "The immediate results must be totally different from those at present obtained, and the after results also, before the operation can be admitted to a place among the legitimate operations of surgery."

Lawson Tait, says: "The proposal to deal with cancer of the uterus by complete removal of the organ meets with my strong disapproval;" and he further states: "My reasons are that its primary mortality must always be heavy, and that the few cases in which the disease does not recur are clearly errors of diagnosis."

Schroeder, of Berlin, now dead, after performing vaginal hysteriotomy on twenty-seven patients, says: "It is not yet to be called satisfactory, especially as far as the question of recurrence is concerned."

Prof. Olshauem, up to 1883 performed this operation twenty-eight times; two of his patients died on the day of the operation; three of septicemia on the second and third days; one of carbolic poisoning on the second day; one of iodoform poisoning on the sixth day, and another also died suddenly of embolism of the pulmonary artery on the sixth day.

Dr. Reeves Jackson, of Chicago, elucidated this question very clearly before the American Gynaecological Society, showing it to be a highly dangerous operation and not productive of reasonable hope of relief.

It has been claimed by the advocates of total extirpation, that when recurrence of the disease does take place, the patient suffers but little toward the end of life, as the spread of the disease is upward in the pelvic cellular tissue, and the patient is saved not only from the dreadful pain, but also from the hemorrhage and ulceration.

While I do not deny that this may occasionally be true, yet I must say that I have never seen it. In cases which I have observed, the pain, foetid discharge, and cachexia, were as pronounced as in those not yet subjected to this operation.

In consequence of the dangers of total hysterectomy, I therefore answer the question: Is extirpation of the cancerous womb a justifiable operation? Most unquestionably in the negative.

This being true, the question naturally suggests itself. Is there any other method of treating uterine cancer, that is at once safer in the technique of operations, and which gives assurance of longer life afterwards? I answer unhesitatingly and unequivocally in the affirmative. In proper cases for operation, and by proper cases for operation, I do not mean those cases in which the disease has progressed to such an extent that the woman who consults you, has already made her own diagnosis—where the ganglia, the parametric tissues, the vagina, and indeed all the surrounding structures are infiltrated and adherent and matted together, or where ulceration is extensive.

As we all know uterine cancer of whatever variety, in its early stages, is a painless disease. We further know, that in at least ninety-five per cent. it begins in, and affects the cervix, and we have no reason to doubt that it is very often, indeed almost always, implanted upon a laceration of the cervix. Although it is accounted by some good authority, Briesky, among them, that it is caused by friction of the cervix on the vaginal floor.

Primitive uterine cancer is very rare in the body of the womb. In the cervix its extension is circumferential and not upwards. Therefore the best and safest manner of its removal is through the vagina—supra vaginal amputation—together with tunnelling to a greater or less extent the body of the womb, as may be indicated or necessary, bearing in mind the paramount necessity of removing every vestige of diseased tissue.

This can be best accomplished by the use of the galvano-cautery, the knife or the hot iron, followed, if need be, by caustics; and which give incomparably better results, both as regards the immediate death rate and the ultimate results.

Time will not permit of a minute descriptive detail in the use of the various instruments and appliances that may be required. But I will venture to tax your patience with a description of a method of operating, which for more than ten years past, I have practiced with great satisfaction, and with far better results than formerly, and to the use of which, I am indebted to the late Angus MacDonald, of Edinborough, Scotland.

After the patient has been fully anaesthetized and placed in a modified lithotomy position, he proceeds to amputate the cervix, which he does with great rapidity, with an ordinary gouge, such as is used in operations for necrosis of bone. He next introduces either a boxwood or vulcanite speculum of large size, and through it applies a Paquelin cautery knife, heated to rather more than a dull red, and burns away all the diseased tissue, many times going up to the fundus, and leaving the body of the uterus a mere shell. Just before completing the operation he allows the heat of his paquelin to become a very dull red and applies it to every part of the wounded surface, which effectually prevents hemorrhage.

It is remarkable, how little pain is endured by patients who have been subjected to the operation in this manner. It would give me pleasure to report cases, but I have already occupied enough of your time. I thank you for your attention.

614 Olive street.

